# A semi-classical approach to the calculation of highly excited rotational energies for asymmetric-top molecules

**DOI:** 10.1039/c6cp05589c

**Published:** 2016-12-09

**Authors:** Hanno Schmiedt, Stephan Schlemmer, Sergey N. Yurchenko, Andrey Yachmenev, Per Jensen

**Affiliations:** a I. Physikalisches Institut , Universität zu Köln , Zülpicher Straße 77 , 50937 Köln , Germany; b Department of Physics and Astronomy , University College London , Gower Street , WC1E 6BT London , UK; c Center for Free-Electron Laser Science , DESY , Notkestrasse 85 , 22607 Hamburg , Germany; d Theoretische Chemie , Bergische Universität Wuppertal , Gaußstr. 20 , 42119 Wuppertal , Germany . Email: jensen@uni-wuppertal.de

## Abstract

We report a new semi-classical method to compute highly excited rotational energy levels of an asymmetric-top molecule. The method forgoes the idea of a full quantum mechanical treatment of the ro-vibrational motion of the molecule. Instead, it employs a semi-classical Green's function approach to describe the rotational motion, while retaining a quantum mechanical description of the vibrations. Similar approaches have existed for some time, but the method proposed here has two novel features. First, inspired by the path integral method, periodic orbits in the phase space and tunneling paths are naturally obtained by means of molecular symmetry analysis. Second, the rigorous variational method is employed for the first time to describe the molecular vibrations. In addition, we present a new robust approach to generating rotational energy surfaces for vibrationally excited states; this is done in a fully quantum-mechanical, variational manner. The semi-classical approach of the present work is applied to calculating the energies of very highly excited rotational states and it reduces dramatically the computing time as well as the storage and memory requirements when compared to the fullly quantum-mechanical variational approach. Test calculations for excited states of SO_2_ yield semi-classical energies in very good agreement with the available experimental data and the results of fully quantum-mechanical calculations.

## Abstract 2

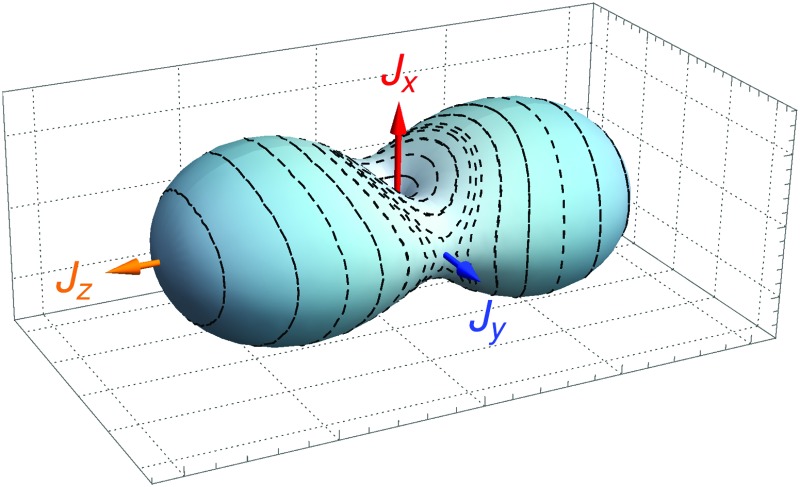
We report a new semi-classical method to compute highly excited rotational energy levels of an asymmetric-top molecule.

## 1 Introduction

The majority of theoretical and experimental studies of molecular properties in highly excited states have focused on probing the vibrational and electronic contributions to the molecular energy. Much less is known about molecules at very high rotational excitation. Several recent theoretical studies have demonstrated that ultrafast rotating molecules open a new avenue for investigating chemical reactivity,^1,2^ low-temperature collisional dynamics,^3–6^ rotational cooling and trapping,^7,8^ and other unusual phenomena occurring for molecules with rotational energies comparable in size to their chemical bond strengths.

Until recently, excited rotational states of molecules were mostly populated in experiments by simply increasing the temperature or using microwave ladder excitation techniques.^9,10^ Owing, for example, to limitations of the temperatures that can be reached in practice, only moderate rotational excitation can be achieved in this manner. With the advent of the molecular optical centrifuge,^1,11^ generated by two powerful counter-rotating ultrafast, oppositely chirped laser pulses, it became possible to prepare and control molecular ensembles at extremely high rotational excitation, even at low temperatures. The centrifuge has been successfully implemented to study the infrared spectrum of CO_2_ in rotational states of very high energy^12,13^ (*J* ≈ 220).

The quantum-mechanical calculation of the rovibronic energies of a polyatomic molecule within the Born–Oppenheimer approximation (see, for example, ref. 14 and references therein) normally involves the choice of a zero-order model, that is, a simplified description of the molecule leading to a Hamiltonian whose eigenvalues and eigenstates are known or easily obtainable. Historically, the zero-order model of choice has been the rigidly-rotating/harmonically-vibrating molecule (see, for example, ref. 15 and references therein) whose rotation is modelled as that of a rigid rotor, and whose vibration is modelled as that of a collection of uncoupled harmonic oscillators. Rotation and vibration are considered as entirely independent, and so the eigenfunctions of the corresponding zero-order Hamiltonian are products of well-known rigid-rotor eigenfunctions and harmonic-oscillator eigenfunctions.^16^ These zero-order eigenfunctions are now taken as basis functions in the calculation of the eigenstates of the actual molecule. That is, the eigenfunctions for the Hamiltonian of the actual molecule are taken as a (truncated) linear combination of the basis functions. This ‘Ansatz’ converts the Schrödinger equation into a matrix equation: each molecular energy is given as an eigenvalue of a matrix representing the Hamiltonian in the chosen basis set,^16^ and the corresponding eigenvector contains the expansion coefficients defining the molecular wavefunction in terms of the basis functions. In the early days of molecular theory, perturbation theory represented the only possibility of diagonalizing the Hamiltonian matrix. The perturbations to the zero-order model are anharmonicity and rotation-vibration coupling. The ensuing theoretical results, developed over many years, constitute an extensive toolbox for the analysis of molecular spectra (again, see ref. 15 and 16 and references therein). The perturbation-theory approach runs into problem in the presence of so-called resonances, when the energy separations between two or more basis states are comparable in size to the matrix elements coupling these basis states. With the advent of high-capacity computers, it became possible to avoid the approximation inherent in the perturbation theory by simply diagonalizing the truncated Hamiltonian matrix numerically. This is known as a variational calculation. Solution of the quantum mechanical problem by means of variational methods provides – compared to the results of perturbation theory calculations – a superior description of molecular energies, in particular for molecules in highly excited states. The variational methods have been developed during the last decade so as to provide theoretical data of a quality that is, at least, close to satisfying the exacting demands of high-resolution spectroscopy.^17–21^ They nowadays make use of zero-order models more sophisticated and physically satisfactory than the rigidly-rotating/harmonically-vibrating molecule. As already mentioned, the matrices to be diagonalized in variational calculations are truncated: the exact solution is only obtained in the limit of an infinitely large basis set which, in practice, we must approximate by a finite number of basis functions. This finite number must be chosen large enough that the computed eigenvalues are converged so that they do not change appreciably when more basis functions are included in the calculation. As described at length in ref. 16, symmetry arguments can be used to facilitate a variational calculation. Nevertheless, the principal impediment to variational calculations, which is most serious for highly excited states, is the substantial size of the basis set required to obtain converged eigenenergies and eigenfunctions, and the ensuing sizes of the Hamiltonian matrices whose diagonalization is often very costly in terms of computation time and storage capacity. Several methods have been developed to tackle the problem of large basis sets by devising appropriate ro-vibrational coordinates,^21^ employing non-direct product contraction techniques^21–23^ and molecular symmetry,^19^ using time-dependent basis functions,^17,24^ or carrying out the matrix diagonalization with low-storage iterative Lanczos^25^ or filter^26–28^ techniques. Notwithstanding these efforts, variational methods remain extremely expensive in terms of computer capacity and, so far, they have been rarely applied to the computation of energies and wavefunctions for highly excited states of molecules with more than three nuclei. However, a recent example of such a calculation (for H_2_O_2_) is given in ref. 29. By means of the symmetry arguments mentioned above,^16^ it can be rigorously shown that the Hamiltonian matrix of an isolated molecule is block diagonal in the angular momentum quantum number *J* (see, for example, ref. 16) and the resulting matrix blocks can be diagonalized independently. This fact can only be exploited with a basis set of eigenfunctions of the angular-momentum operator  *J* ^2^, and the well-known symmetric-top-eigenfunctions^16^ |*J*,*k*,*m* are normally used. The Hamiltonian matrix blocks are diagonal in the projection quantum number *m* (= –*J*, –*J* + 1, …, *J* – 1, *J*) and the matrix elements do not depend on *m*,^16^ so one can do a single calculation for, say, *m* = 0 for a given value of *J*. However, as *J* increases we must take into account increasingly more values of *k* (= –*J*, –*J* + 1, …, *J* – 1, *J*); there are 2*J* + 1 values of *k* for *J* given. Hence, the dimension of a Hamiltonian-matrix block increases proportionally to 2*J* + 1 and so, for increasing *J*, the matrix dimension will eventually reach a limit at which diagonalization is no longer feasible with the available computers.

With the aim of avoiding the “high-*J*-catastrophe” just described, we explore in the present work an alternative approach to the calculation of energies for highly excited rotational states of molecules. It is inspired by the fact that at high rotational excitation, as the rotational angular momentum attains ‘macroscopic’ values vastly larger than *ħ* = *h*/2π (where *h* is Planck's constant), the behavior of the molecule changes from being quantum mechanical to being classical.^30^ This is known as the correspondence principle. Correspondence-principle-inspired, theoretical descriptions of molecular rotation (sometimes called semi-classical theory) were developed already in the 1970s and 80s^31–33^ but, with a few exceptions,^33–38^ they have faded into oblivion. In a semi-classical approach, the rotation is described in terms of classical periodic trajectories on a so-called rotational energy surface (RES). The RES is a function of classical angular momentum components (*J*_*x*_,*J*_*y*_,*J*_*z*_) measured along molecule-fixed axes *xyz* (see, for example, ref. 16). The function values are obtained as molecular rotation-vibration energy values for the vibrational state under study and for given fixed values of (*J*_*x*_,*J*_*y*_,*J*_*z*_).^39–41^ RES theory has also been developed to take into account the effect of tunneling between two equivalent rotational trajectories by including classically forbidden complex periodic orbits in phase space.^36^ One of the purposes of the present work is to review and generalize previous semi-classical RES theory by couching it in the path integral formalism.^42^ Another – which is described for the first time here – is the generation of the RES by in fully quantum mechanical calculations with the TROVE program.^19,21^

A central concept of the path integral formalism is the quantum mechanical propagator which, in its original derivation, gives the probability of finding a particle, whose position was given by the coordinate value *q*_i_ at the time *t* = 0, with the position coordinate value *q*_f_ at a later time *t*. The propagator can be expressed as an integral, which conveniently contains the Lagrangian (or the Hamiltonian) of the underlying physical system. In very general terms, one can write the propagator *K*(*q*_*i*_,*q*_*f*_,*t*) as:1
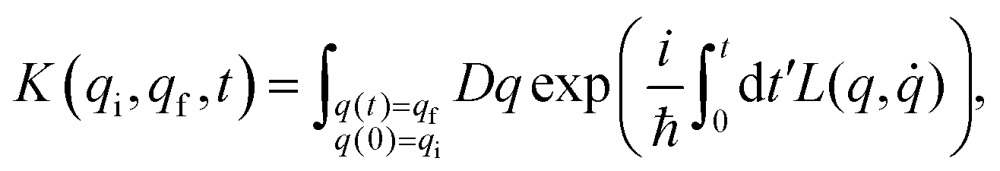
where *p* is the momentum of the particle and *L* = *mq* ^2^ – *V*(*q*) represents the Lagrangian with the potential energy function *V*(*q*). The functional measure is defined as 
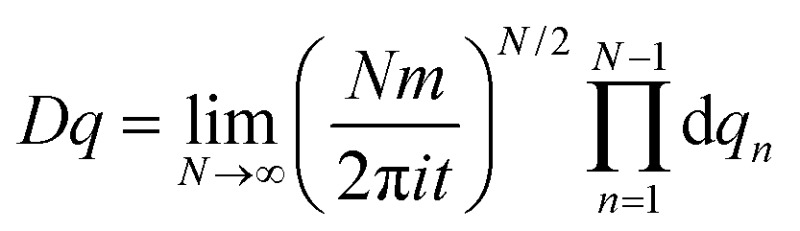
.^30^ The physical interpretation is rather intuitive: the probability of the particle getting from *q*_i_ to *q*_f_ during the time *t* is given as an integral over all possible paths from *q*_i_ to *q*_f_, where each individual path is weighted by its action, equal to the time integral over the appropriate Lagrangian function along the path in question. The action attains its maximum value for the classical path but, in contrast to usual classical mechanics, the path integral takes into account all other, less probable paths which makes it a suitable tool for describing quantum mechanical processes. The strategy of weighting the paths by their action is the starting point of the semi-classical description. Since the path integral can also be used in a perturbative context, one can allow only for “small” fluctuations around the classical path and hence derive a semi-classical approximation of the propagator. It is “the ideal starting point for semiclassical calculations” (ref. 43, p. 262), but is nowadays also used as the fundamental basis of quantum field theory, which is highly important in many fields of single particle and many-body physics and chemistry. In the approach of the present work, we use the density of states – a physical observable – as the starting point for the discussion of the semi-classical approximation for the energy levels of the rotating molecule. The density of states is calculated from the Fourier transform of the propagator. As the rigorous derivation of the density of states is beyond the scope of the present work, we refer to textbooks such as ref. 30 and 43. The density of states exhibits poles at the quantized energy levels and these poles can be calculated using the semi-classical RES by identifying certain periodic orbits on it. These paths are selected by symmetry arguments and can be calculated by considering the operations of the molecular symmetry group,^16^ which can be correlated with motions on the RES.

The resulting, new method describes the effect of tunneling in a more rigorous and natural way involving a molecular symmetry group^16^ analysis; this yields results equivalent to the original ones from ref. 36. Another novel aspect of the proposed method is that the underlying RES is obtained quantum mechanically by means of variational theory as implemented in computer program TROVE.^19,21^ This is probably the most important progress made in the present work. Indeed, it is not the first time that quantum-mechanically calculated RES's have been subjected to classical analysis,^44,45^ but we make a further development here in that we describe the tunneling effects just mentioned. In earlier works, effective Watsonian-type Hamiltonians or (semi-)classical vibrational models have been used to determine the RES.^33,46,47^ The combination of the high accuracy first-principles quantum mechanical treatment of the (excited) vibrations by TROVE, and the semi-classical treatment of the rotation (including the tunneling effects), makes the method of the present work a versatile tool for the understanding of ro-vibrational molecular dynamics at high rotational excitation. This is one of the chief points of the present work: our calculations directly connect the (quantized) rotational energies of a single molecule at large rotational speed to particular, unambiguously defined paths on the vibrationally averaged RES. This induces a clear understanding of this high-speed motion, in contrast to pure quantum studies, where the eigenvalues of a Hamiltonian matrix provide energies more accurate than those obtained semi-classically, but where the motions associated with the quantum-mechanical energies can only be understood (and that only to a certain extent) by complicated visualizations of the wavefunctions. The semi-classical approach allows an intuitive and definitive interpretation of the molecular states in terms of quasi-classical motions.

For the proof-of-principle numerical tests of the present work we have chosen the SO_2_ molecule since its energy level pattern has been characterized to high angular momentum values (*J* ≤ 80), both experimentally^48^ and in fully quantum-mechanical, variational calculations.^49^ For several vibrational states of SO_2_, we have calculated semi-classical rotational energies which show very good accuracy when compared to experimental data or with the results of full quantum mechanical calculations. By comparisons with experiment we must take into account that only the *A*_1_ and *A*_2_ states of SO_2_ – with symmetry group *C*_2v_(M)^16^ – are allowed by nuclear spin statistics, while *B*_1_ and *B*_2_ rovibrational states are forbidden.^16^ Our calculations do not as yet consider spin statistics.

The paper is organized as follows. In Section 2 we formulate the general semi-classical treatment of molecular rotational motion using the path integral approach. The calculation of the RES for a general molecule by means of TROVE is presented in Section 3. In Section 4 we report semi-classical calculations of the rotational energies for the SO_2_ molecule and assess the accuracy of the results. Section 5 finally provides a summary and a discussion of the perspectives for future studies.

## 2 The semi-classical approach

At large angular momentum, we expect the rotational dynamics to be governed by the classical Euler–Lagrange equations of motion. Accordingly, a path integral approach – being a versatile tool for studying classical mechanics – is well suited for solving these equations. Assuming the actual motion to take the form of small fluctuations around the classical path, we take the density of states *ρ*(*E*) to be given by the semi-classical expression long known as “Gutzwillers trace formula”^42^
2
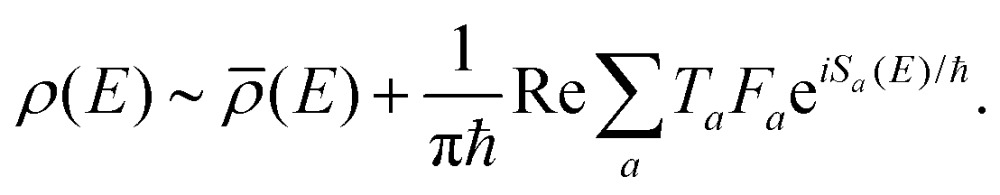
Here, we sum over periodic orbits *a* of the classical system weighted by the respective action *S*_*a*_ = **p**_*a*_d**q**_*a*_, the period *T*_*a*_ and a stability amplitude *F*_*a*_, which here includes the Maslov index. The quantity *ρ̄*(*E*) is the smooth part of the density of states, calculated as the purely classical contribution. For a detailed mathematical description of the semi-classical approach to obtaining the density of states in the path-integral formalism, we refer the reader to ref. 30, 50 and 51.

In rotationally resolved molecular-spectroscopy studies, the actual energy levels are of principal interest. In the approach of the present work, they can be determined at the poles of the oscillatory part of *ρ*(*E*). Furthermore, the energy levels are labeled by their symmetry species (irreducible representations) in the appropriate symmetry groups^16^ of the molecular Hamiltonian. With the symmetry labels we can obtain, for example, selection rules for state-to-state transitions. Projecting the density of states in eqn (2) onto the space of states belonging to one particular irreducible representation *Γ*_*m*_, we obtain the symmetry reduced oscillatory part of the density of states:^52^
3
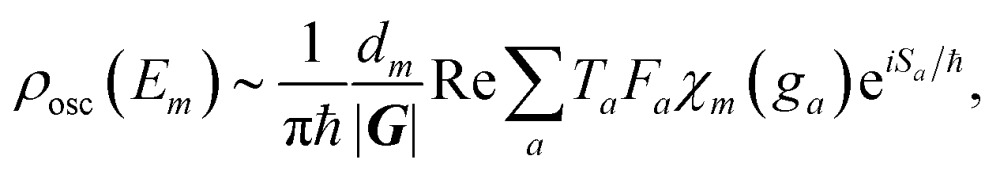
where *d*_*m*_ is the dimension of *Γ*_*m*_, *χ*_*m*_ the character of the related matrix representation of the symmetry group element *g* and 
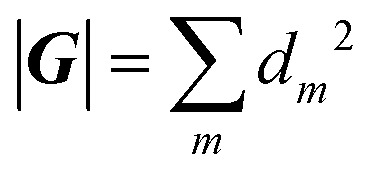
 denotes the number of elements in the symmetry group G. Furthermore, the orbits to be summed over are now periodic in the symmetry reduced phase space and their initial and final points are related by a symmetry operation *g*_*a*_, which is the unit element, ensuring the periodicity of the orbit. This unit element can be composed of numerous symmetry elements.

To include also quantum fluctuations superimposed on the classical motion, we allow for so-called beam splitting, where the paths get reflected or transmitted at their classical turning points in phase space.^36,53^ The transmitted – and hence classically forbidden – paths are described in phase space by a Wick-rotation^54^ to imaginary time so that the momentum becomes complex. Consequently, the related action is imaginary and produces an exponential damping factor in the density of states (*cf.* the discussion of instantons in double-well potentials in ref. 30). We introduce the well-known WKB reflection and transmission amplitudes^46,55^ *r* and *t* for the quantum fluctuations and in terms of these (see Appendix for their definitions), the oscillatory part of *ρ*(*E*_*m*_) is formally given by:4
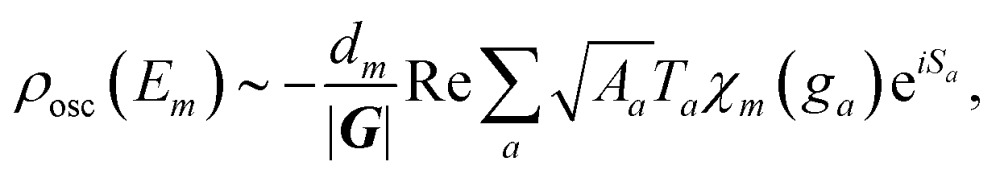
where 
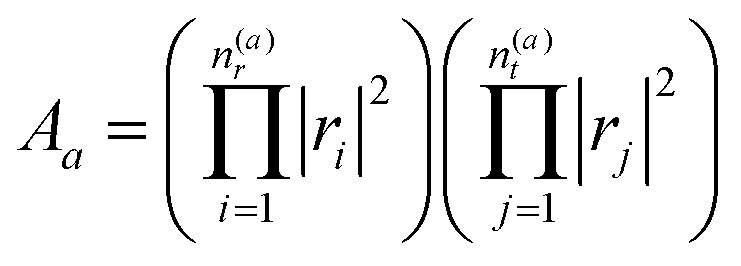
 describes the product of *n*(*a*)*r* reflections and *n*(*a*)*t* transmission probabilities, characterized by *r*_*i*_ and *t*_*j*_ respectively. An appropriate description of the paths, combined by real (*i.e.*, classically allowed) and tunneling (*i.e.*, classically forbidden) segments is made by carefully choosing the symmetry group elements describing the different segments and combining them in a sequence corresponding to the sequence of factors in *A*_*a*_. Thus, each path is characterized by a sequence of the type *r* ^*a*–1^ *t*e^*iaS*^, where *a* is the number of real segments between the one transmission and the end of the path (*cf.*eqn (11) in ref. 46) and the action *S* is identical for all *a*. The sum over all possible combinations of, *e.g.*, the number of real segments between two transmissions or over the number of transmissions in total, is initially rather involved but, fortunately, it can be reduced to a geometric series, and a relatively simple expression is obtained:5
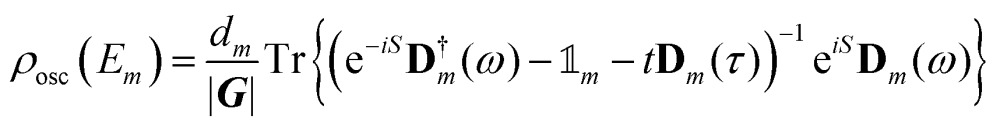
where *ω* and *τ* parametrize the real and tunneling symmetry elements, respectively, and the **D**_*m*_ are the representation matrices constituting *Γ*_*m*_ (
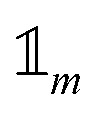
 is the unit element of *Γ*_*m*_). The poles, *i.e.* the energy levels, are now found by the determinant equation:6
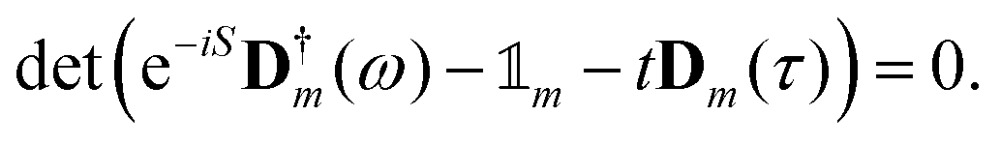
Here, *ω* and *τ* describe the symmetry operations parameterizing the real and tunneling paths, respectively. Equipped with this equation and the molecular symmetry group, it becomes possible to find the quantized energies of any well-defined rotational energy surface. In the following section, we will apply this strategy to the test molecule SO_2_ whose energies are well known, both experimentally and theoretically, up to large values of the angular momentum quantum number *J*.

## 3 The rotational energy surface

For a single vibrational state, the semi-classical approach initially requires a RES^35,36^ obtained, as mentioned above, by replacing the molecule-fixed components of the angular momentum operator  Ĵ = (*Ĵ*_*x*_,*Ĵ*_*y*_,*Ĵ*_*z*_) by their classical, time-dependent analogons J = (*J*_*x*_,*J*_*y*_,*J*_*z*_). This defines a Hamiltonian function *H* depending on (*J*_*x*_,*J*_*y*_,*J*_*z*_) and we make allowance for quantum mechanics in that we fix the length of J to be |J|^2^ = *J*(*J* + 1)*ħ* ^2^. This function is then used to calculate the action 
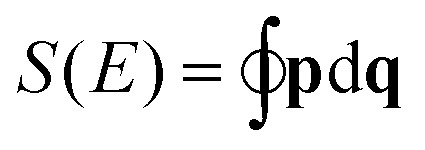
 of the closed orbits, where **p** is simply the solution of *H*(*p*,*q*) = *E*. The trajectories themselves are found on the RES and hence it is the fundamental starting point for the approach described in Section 2 when applied to the rotational problem.

The RES has been successfully used to explain qualitatively the emergence of rotational energy clusters for large *J* quantum numbers.^33^ In order to provide proof of principle, in the present work we focus on a simple molecule with no rotational-energy clustering. The absence of energy cluster formation requires that the RES does not change topologically as *J* increases. We have found that the sulfur dioxide molecule SO_2_ satisfies this requirement and, in addition, its rotation-vibration energy level pattern is well characterized up to high *J* values.^48,49,56^ For these two reasons, we have chosen to validate our approach by computing the rotational energies of SO_2_.

To explain how we calculate the RES, we consider now an initially general ro-vibrational Hamiltonian for an *N*-atomic molecule defined in terms of the 3*N* – 6 generalized vibrational coordinates *ξ*_*k*_, the conjugate momentum operators *p*_*k*_ = –*iħ*∂/∂*ξ*_*k*_, and three angular momentum operators *Ĵ*_*x*_, *Ĵ*_*y*_, *Ĵ*_*z*_
7
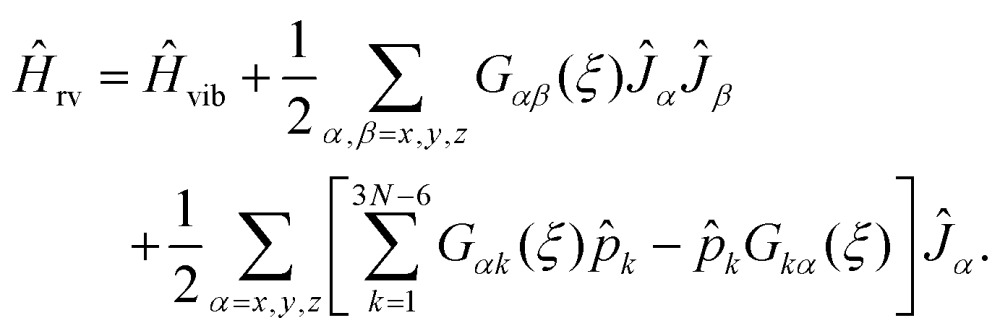
Here, *Ĥ*_vib_ denotes the purely vibrational Hamiltonian and includes the vibrational kinetic energy operator, the Born–Oppenheimer potential energy function and the pseudo-potential terms (see, for example, ref. 19 and 21). The remaining two terms (involving the *Ĵ*_*α*_ operators) describe the rotational kinetic energy and the ro-vibrational (Coriolis) coupling, respectively. In eqn (7), the quantities *G*_*αβ*_(*ξ*) and *G*_*αk*_(*ξ*) are functions of the vibrational coordinates *ξ* ≡ *ξ*_1_,…*ξ*_3*N*–6_. Depending on the definition of *ξ* and the choice of the molecule-fixed Cartesian axes, *G*_*αβ*_(*ξ*) and *G*_*αk*_(*ξ*) can be evaluated analytically or numerically on a grid^20^ or as a power series expansions around a suitable reference structure.^19,21^

As mentioned above, the rotational energy surface, required for determining the paths in the semi-classical approach discussed here, is now defined by replacing in eqn (7) the operators (*Ĵ*_*x*_, *Ĵ*_*y*_, *Ĵ*_*z*_) by their classical counterparts (*J*_*x*_, *J*_*y*_, *J*_*z*_), here defined as8
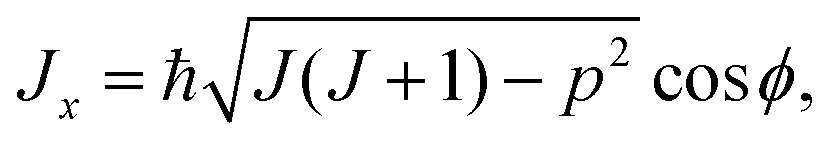

9
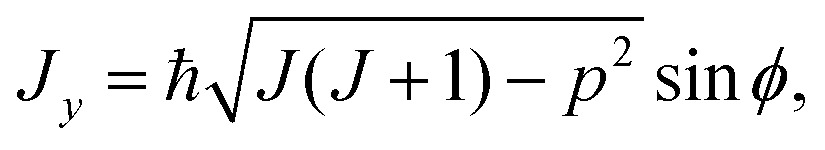

10 *J*_*z*_ = *ħp*,

where, for convenience, we have introduced
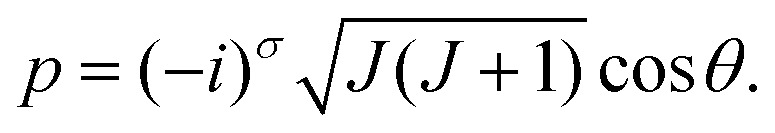
Here, *θ* and *φ* are usual spherical coordinates defining the direction of J in the molecule-fixed axis system *xyz*, and *σ* = 0 or 1. A *σ*-value of 0, with *J*_*z*_ being real, corresponds to a real, classically allowed path, whereas a value of 1, with an imaginary value of *J*_*z*_, corresponds to a classically forbidden tunneling path (see Section 2). Following ref. 33, also in the *σ* = 1 tunneling case we calculate (*J*_*x*_^2^ + *J*_*y*_^2^)/*ħ* ^2^ as *J*(*J* + 1) – *p* ^2^ [eqn (8) and (9)], in spite of the fact that in this case, this quantity is larger than *J*(*J* + 1).

We make only the replacement (*Ĵ*_*x*_, *Ĵ*_*y*_, *Ĵ*_*z*_) → (*J*_*x*_, *J*_*y*_, *J*_*z*_) in *Ĥ*_rv_; we do not change *Ĥ*_vib_. Thus, we obtain a Hamiltonian that retains in full the quantum mechanical description of the vibrational motion while it depends parametrically on the two rotational variables (*θ*, *φ*) and on the angular momentum quantum number *J*. To our knowledge, this is the first time that RESs have been generated for different vibrational states in a full quantum solution.

We use TROVE to solve the ro-vibrational Schrödinger equation at fixed values of the parameters *θ* ∈ [0,π], *φ* ∈ [0,2π], and *J*. Each of the resulting vibrational eigenvalues *E*_1_,…*E*_*n*_ of the Hamiltonian provides a point on the RES for the vibrational state in question, *i.e.* RES_*i*_(*θ*,*φ*) = *E*_*i*_. Following this procedure, the RESs for several vibrational states and several values of *J* may be obtained on a grid of (*θ*, *φ*) coordinates by solving the vibration-only eigenvalue problem for each grid point. One may think that the task of finding the RES by repeatedly solving the eigenvalue problem for thousands of points is quite expensive and unlikely to be very competitive with the full variational treatment. This is partly true for low values of *J* but, as *J* increases the steep (2*J* + 1)^3^ computational scaling of the full variational treatment is quickly outstripped by the RES calculation, which involves the diagonalization of matrices with dimensions independent of *J*.

In the present work we employ two methods for solving the vibrational problem: the Watson-type effective-Hamiltonian method,^57^ and the rigorous variational method, as implemented in the computer program TROVE.^19,21^ The purpose of considering the former method is somewhat academic; the values of the effective-Hamiltonian parameters are normally determined by least-squares fitting to experimental spectroscopic data, and the amount of such data available is typically limited to rotational states at moderate excitation in a few vibrational states. The extrapolation to high rotational excitation is problematic. On the contrary, the variational approach TROVE is general and can be applied to molecules of an arbitrary structure. It relies on the *ab initio* or spectroscopically refined potential energy surfaces and makes no approximative assumptions about the kinetic energy operator. The effects of anharmonicity and Coriolis coupling are intrinsically included in a TROVE-generated RES. For more details about the TROVE approach, the interested reader is referred to ref. 19 and 21.

## 4 The semi-classical energies of the distortable rotor

**Fig. 1:**
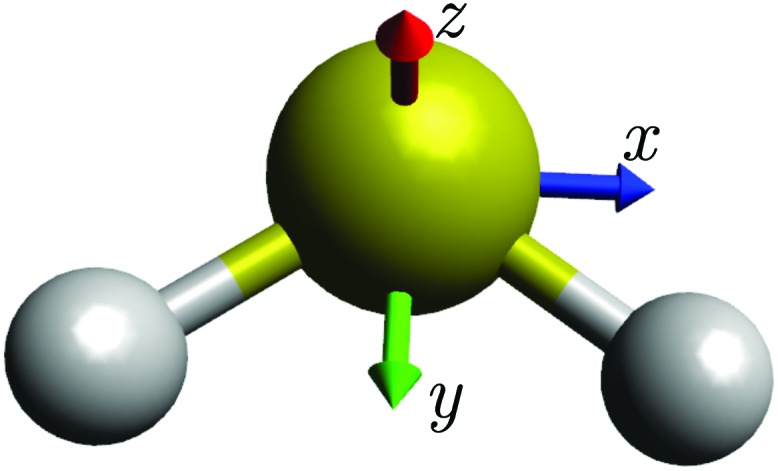
The sulfur dioxide molecule SO_2_ at equilibrium with the molecule-fixed axis system *xyz* attached. The molecule is in the *xy* plane.

To our knowledge, Robbins *et al.* ^36,46^ have provided, more than twenty-five years ago, the very first example of the semi-classical, path-integral method discussed here being applied to a molecule. They treated the rotational dynamics of SF_6_, described by the (effective) Hecht Hamiltonian^58^ with octahedral symmetry. Their results showed good agreement with energy levels determined in quantum mechanical calculations. We will first investigate how the semi-classical method performs when applied with a RES obtained from a Watson-type effective Hamiltonian, and then apply it with a TROVE-generated RES.^19,21^ Our results suggest that it is generally useful in conjunction with any Hamiltonian adequate for describing the molecular energy levels. It does not require the Hamiltonian to depend on the angular momentum operators in any particular way, in contrast to the approach of ref. 36 which used the “Hecht Hamiltonian” for SF_6_. It is not limited to any power of the perturbation expansion used to obtain the Watson-type effective Hamiltonian and it can be applied to a vibrationally averaged RES as done in the TROVE approach. As already mentioned, our test case is the sulfur dioxide molecule SO_2_ (see Fig. 1), which is not expected to exhibit rotational-energy cluster formation, and for which there are extensive data available for comparison.

**Fig. 2:**
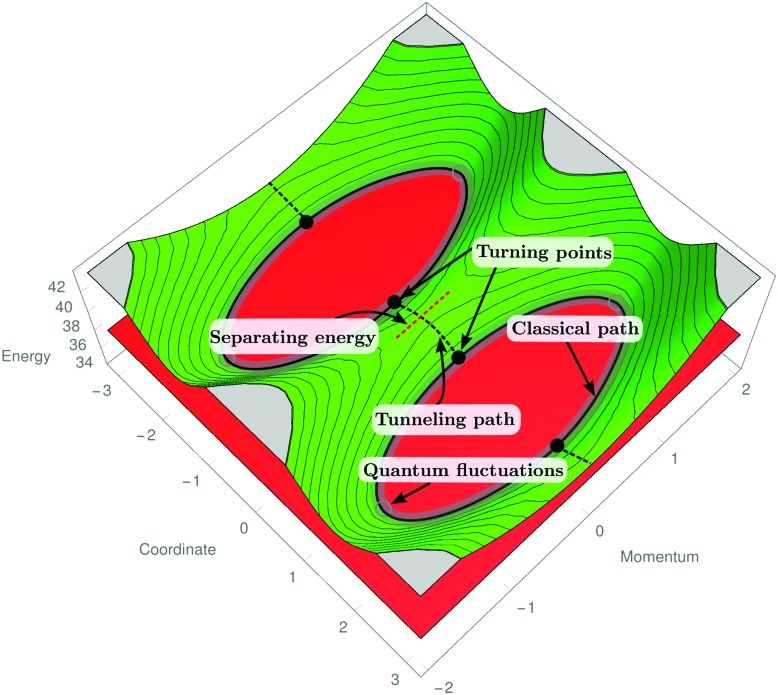
A schematic phase-space view of the SO_2_ rotational energy surface. The RES obtained for a fixed *J* value is shown as a green surface, while the red surface represents the constant energy. The real, classically allowed paths are the intersections of these two surfaces. The separating energy *E*_s_ governs the transition from the quantization axis considered here to an alternative one, since for energies *E* > *E*_s_ no closed path is possible.

In the absence of rotational energy clusters, the RES for an asymmetric rotor generally has two extrema corresponding to two main axes of rotation. These axes give rise to the largest and smallest moment of inertia, respectively. A radial plot of the RES shows the extrema explicitly and such plots, compared with constant-energy spheres, are used to determine the classical solutions of the Euler–Lagrange equations of motion.^59^ In particular, the intersection of the RES with a constant-energy sphere represents a classical path on which the angular momentum and the energy are both conserved. This corresponds to the physically intuitive picture of a rotating body with a main axis of rotation that precesses around the angular momentum vector (whose direction and length are conserved, *i.e.*, time-independent). In the semi-classical approach, we project the RES onto the two main axes of rotation (*cf.* Fig. 2). Inspecting the classical paths corresponding to these two projections, we notice that, depending on the energy, there is a single axis around which the path is closed. The projection, in itself, defines a coordinate and a conjugate momentum. This conjugate momentum is paramount for carrying out the semi-classical analysis; the action for the path in question is calculated in terms of the angular momentum along this path. In particular, for the paths closing around the *J*_*x*_ axis (called *J*_*x*_ quantization in Table 1), the *J*_*z*_ axis is chosen to define the momentum *p*, whereas in the *J*_*z*_ quantization case, *J*_*x*_ = *p*.

**Table 1:** Parameterizations of the real and tunneling paths of SO_2_ obtained for the quantization axes *x* and *z* and for the four possible irreducible representations of *C*_2v_(*M*) (see text). The quantization conditions obtained from eqn (12) require the listed quantities to vanish for an integral value of *n*. Here, *δ* is the phase shift at a classical turning point and *S* represents the tunneling action. For exact definitions see appendix and ref. 46

	*J*_*x*_-Quantization	*J*_*z*_-Quantization
*ω*	(12)* ≙ *R*π*x*	E* ≙ *R*π*z*
*τ*	(12) ≙ *R*π*y*	(12) ≙ *R*π*y*

*A*_1_	*δ* + *S* – (–1)^*J*^ tan^–1^ e^*S*^ – 2*n*π	*δ* + *S* – (–1)^*J*^ tan^–1^ e^*S*^ – 2*n*π
*A*_2_	*δ* + *S* – (–1)^*J*^ tan^–1^ e^*S*^ – (2*n* + 1)π	*δ* + *S* – (–1)^*J*^ tan^–1^ e^*S*^ – (2*n* + 1)π
*B*_1_	*δ* + *S* + (–1)^*J*^ tan^–1^ e^*S*^ – 2*n*π	*δ* + *S* + (–1)^*J*^ tan^–1^ e^*S*^ – (2*n* + 1)π
*B*_2_	*δ* + *S* + (–1)^*J*^ tan^–1^ e^*S*^ – (2*n* + 1)π	*δ* + *S* + (–1)^*J*^ tan^–1^ e^*S*^ – 2*n*π

In order to find the poles of the density of states, the parameterization of the paths in terms of the real and the tunneling segments is crucial. It depends on the chosen projection axis and is done using two different rotation operators (*cf.* Table 1). The two operations *ω* and *τ* determine the path and hence they define the periodic orbits. Periodicity is ensured by the use of a combination of symmetry elements, so that their successive application, as described in Section 2, defines a path on the RES starting and ending in the same point. This is shown in Fig. 2, where classical and tunneling paths are displayed on the (projected) RES. As stated above, only for one choice of the projection these closed paths exist and hence, the two choices of (*ω*,*τ*) are mutually exclusive.

If a symmetry group is specified for the molecule under study, we follow the strategy of Robbins *et al.* ^46^ and determine the poles of the density of states [eqn (4)] by solving eqn (6). For SO_2_, the molecular symmetry group^16^ is the simple, four-element group

11  *C*_2v_(*M*) = {*E*,(12), E*, (12)*}

where^16^ *E* is the unit operation, (12) is the interchange of the two O nuclei, E* is the inversion operation and (12)* = (12)E*. The *C*_2v_(*M*) group has four irreducible representations *A*_1_, *A*_2_, *B*_1_ and *B*_2_ which are all non-degenerate (see Table A-5 of ref. 16). Thus, the quantized energy levels *E* of the asymmetric rotor are straightforwardly determined as the solutions of

12 e^–*iS*(*E*)^*χ*_*m*_(*ω*) – *r*(*E*) – *t*(*E*)*χ*_*m*_(*τ*) = 0,

where the action *S*(*E*), and the reflection and transmission amplitudes (*r*(*E*) and *t*(*E*), respectively), are functions of the energy. For the two different choices of projection axes (*x* and *z*) and the four different symmetry species *A*_1_, *A*_2_, *B*_1_ and *B*_2_, we get distinct quantization rules summarized in Table 1. The table lists also the so-called equivalent rotations of the elements in *C*_2v_(*M*). For each group element, the equivalent rotation has the same effect on the rotational coordinates as the group element itself (see ref. 16). In Table 1, the equivalent rotations are expressed in terms of operators *R*π*α*, rotations of 180° about the *α* (= *x*, *y*, *z*) axis (see Fig. 1). Thus, for example, (12)* has the equivalent rotation *R*π*x*, a rotation of 180° about the *x* axis.

Note that in both projections, the tunneling paths are described by the same symmetry element (12), producing a reversal of the main axis of rotation and thus a reversal of the handedness of the respective rotation. Furthermore, in the case of zero tunneling probability, doubly degenerate states of symmetry *A*_1_ ⊕ *B*_1_ or *A*_2_ ⊕ *B*_2_ ensue for the *J*_*x*_ quantization, whereas for the *J*_*z*_ quantization axis, the degenerate states have symmetries of *A*_1_ ⊕ *B*_2_ or *A*_2_ ⊕ *B*_1_.

We now apply our method to the asymmetric-top molecule SO_2_. The RESs for different vibrational states of SO_2_ are generated using two methods, the Watson-type effective Hamiltonian and the variational approach TROVE. The parameter values of the effective Hamiltonian have been determined by fitting to experimental spectroscopic data. In the TROVE calculations we employed an accurate potential energy surface for the ground electronic state of SO_2_, computed *ab initio* and refined in fittings to experimental data.^49,56^ During the course of the present work, new *ab initio* potential energy surfaces for the *X* ^1^ *A*_1_ and *C* ^1^ *B*_2_ (2^1^ *A*′) electronic states of SO_2_ were reported by Kłos *et al.* ^60^ We prefer to use here the ground state surface from ref. 49 and 56 since it was optimized in a least-squares fitting to experimental data and this produces, in our experience, the highest accuracy.

Following the general treatment discussed above, we implement our quantization procedure through a step-by-step strategy: (i) choose a quantization axis, dictated by the energy such as to project the RES onto a one-dimensional problem *H*(*p*,*q*); (ii) solve *H*(*p*,*q*) = *E* for *p* in order to calculate the action of the real paths (real solutions) and the tunneling segments (imaginary solutions); (iii) calculate the left hand side of eqn (12) for various energies; (iv) locate the energy values for which eqn (12) is satisfied. The semi-classical results obtained from the RESs generated using TROVE and the Watson-type, effective Hamiltonian approach are henceforth referred to as TROVE-RES and Watson-RES, respectively.

**Fig. 3:**
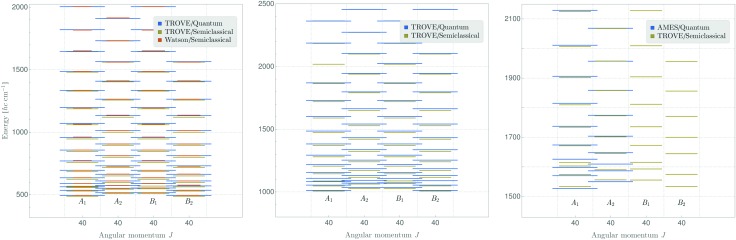
Rotational energies of SO_2_ for *J* = 40 in the vibrational ground state (left panel) and in the *ν*_2_ state (center panel), calculated as discussed in the text. The symmetry species of the states in *C*_2v_(*M*) are indicated. In the right panel, we show also 2*ν*_2_ levels, where we compare to recent quantum calculation of ref. 49, where only the Pauli-allowed species of *A*_1_ and *A*_2_ symmetry were treated. In that comparison, we have subtracted a constant offset of 10 cm^–1^ from the semi-classical values. At present, we have no explanation for this offset.

Fig. 3 (left panel) compares *J* = 40 rotational energy values for the vibrational ground state of SO_2_ obtained in a fully quantum-mechanical TROVE calculation with values resulting from the semi-classical method of the present work. The right panel of the figure makes an analogous comparison for the *ν*_2_ vibrational state for which, however, no Watson-type effective rotational Hamiltonian was used. In both cases, the semi-classical results agree with the fully quantum-mechanical ones to within a few cm^–1^. Note that the results presented here include all ro-vibrational eigenstates, also the ones forbidden by Bose–Einstein statistics.

**Fig. 4:**
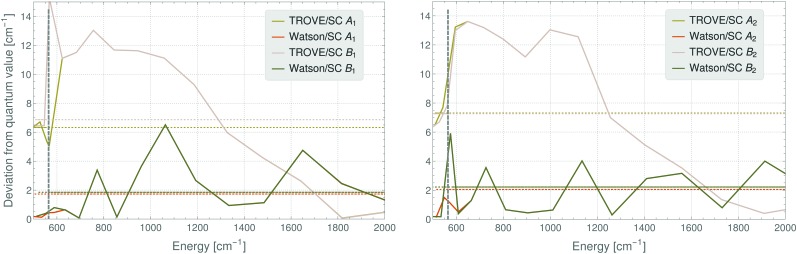
Absolute deviations (in cm^–1^) between semiclassical and fully quantum-mechanical TROVE values for the rotational energies in the vibrational ground state of SO_2_. Very similar patterns are obtained for other vibrational states. The dashed grey line indicates the change of the quantization axis inducing near-degeneracy of state pairs (see text).

**Fig. 5:**
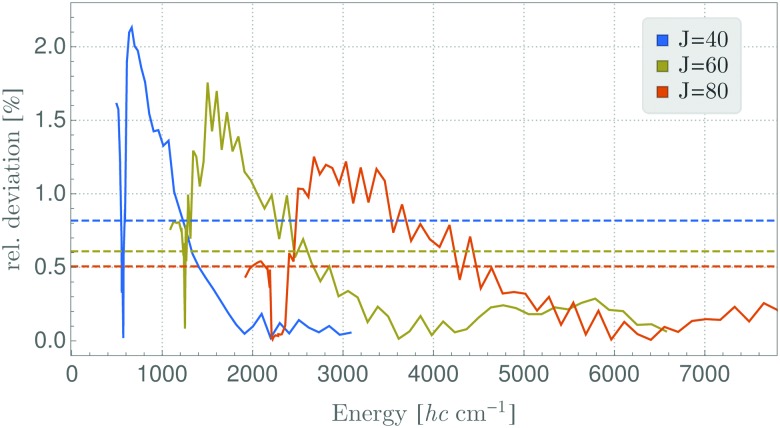
Relative deviation (*E*_SC_ – *E*_QM_)/*E*_QM_ of semi-classical energy levels *E*_SC_ calculated using TROVE generated RES for the vibrational ground state and according full quantum-mechanical energies *E*_QM_. The deviation is shown in percent and the dashed lines represent the mean of this deviation.

A detailed inspection of the deviations between the semi-classical results and the fully quantum-mechanical energies shows that these are larger for the TROVE-RES than for the Watson-RES results (Fig. 4). In addition, the deviation pattern seen in Fig. 4 for the *A*_1_ ro-vibrational energies in the vibrational ground state of SO_2_ is found to be repeated for the other symmetry species, and for other vibrational states. A possible, physical reason for the large deviations of the two models could be that the TROVE calculation of the RES, being perturbation-theory-free and involving high-level vibrational wavefunctions with anharmonicity effects included, is conceptually different from, and much improved relative to, the approach based on a Watson-type effective rotational Hamiltonian obtained with perturbation theory and harmonic-oscillator vibrational basis functions. We think that particularly this point needs further investigation; a full understanding of the vibrationally averaged RES is imperative for the calculation of energy levels by semi-classical methods, especially when these energy levels are so highly excited that, for reasons of computer capacity, they cannot be computed by fully quantum-mechanical methods. As a first step in this direction, we have calculated, by semi-classical methods, energy levels from a TROVE-generated RES for angular momentum quantum numbers of *J* = 40, 60, and 80. We present in Fig. 5 the deviations of these values from the full quantum-mechanical values obtained in complete TROVE calculations. In order to facilitate the comparison in the different energy ranges corresponding to the increasing values of *J*, we show in the figure the relative deviations, *i.e.*, (*E*_SC_ – *E*_QM_)/*E*_QM_, where *E*_SC_ and *E*_QM_ are the semi-classical and quantum-mechanical energy values, respectively. The results for *J* = 80 are calculated only up to energies of 7800 cm^–1^ above the ground state. For higher energies and higher *J* quantum numbers, computer limitations preclude the quantum-mechanical calculations. The results in Fig. 5 clearly indicate that the relative deviation decreases with (i) increasing energy as well as with (ii) increasing *J* quantum number. Therefore, we expect semi-classical studies of even higher *J* quantum numbers, where the quantum calculations are unfeasible, to be accurate.

## 5 Conclusion

**Fig. 6:**
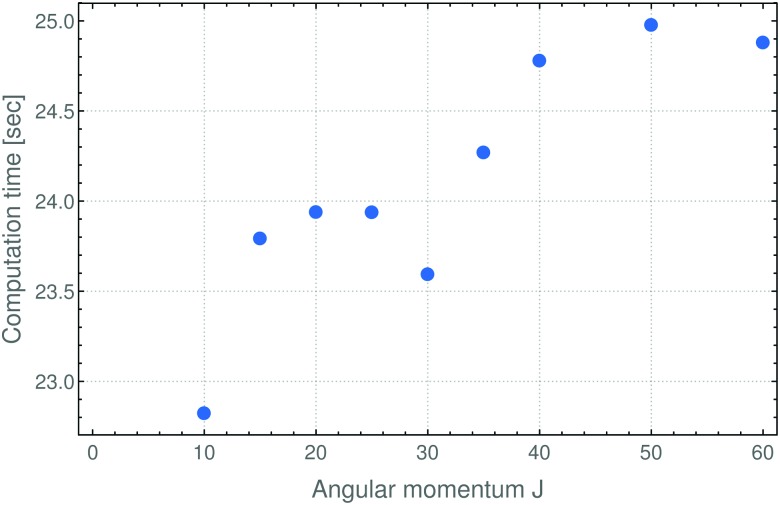
Computation times for semi-classical, Watson-RES calculations of the rotational energies for SO_2_ in its vibrational ground state (see text). The computations were done on a standard PC. The computation time increases roughly linearly with the angular momentum quantum number *J*. The times needed for the corresponding TROVE-RES calculations are about three times larger than those given here, since the TROVE-RES is not parameterized, but also in this case, the computation time is approximately linearly dependent on *J*.

In summary, in the present work we have reformulated, in terms of a path-integral formalism, the semi-classical approach to determining molecular energy levels for states with large angular-momentum values. We have combined various, previously known techniques including Gutzwillers trace formula,^42^ and symmetry analysis based on the molecular symmetry group,^16^ and the isomorphic group of equivalent rotations.^16^ Furthermore, we have introduced a new robust approach to generate the rotational energy surface for vibrationally excited states variationally, by solving the vibrational Schrödinger problem on a grid of the angles (*θ*,*φ*) that define the direction of the angular momentum vector J in the molecule-fixed axis system *xyz*. Thus the solution of the vibrational problem is fully quantum-mechanical, only the highly excited rotational dynamics are treated semi-classically. We have done calculations generating the rotational energy surface in the TROVE approach^19^ and in the perturbation-theory approach leading to the Watson-type effective rotational Hamiltonian. These proof-of-principle calculations were done for the SO_2_ molecule, which has been studied spectroscopically for a long time and for which observations have been made for the angular momentum quantum number *J* < 70. When compared to energy values obtained in purely quantum-mechanical calculations, the semi-classical results are in broad agreement with these, the typical deviations being a few cm^–1^ (see Fig. 3 and 4). That is, currently the semi-classical energy values are not sufficiently accurate to satisfy the exacting demands of high-resolution molecular spectroscopy, but the present work constitutes a first step towards determining molecular energies at high rotational excitation. There are two main advantages of our new approach: Firstly, the computing time for calculating the energies for a given *J*-value is largely independent of *J*; it is mostly dependent on the efficiency of producing explicit rotational energy surfaces by quantum-mechanical solution of the vibrational problem. All steps of quantizing the rotational energy surface are done very efficiently and the computing time scales only with the range of energy required for a given RES (see Fig. 6). By contrast, as mentioned above, the computing time for a given *J*-value typically scales as (2*J* + 1)^3^ in fully quantum-mechanical, variational calculations of rotation-vibration energies. Secondly, the rotational energy surface allows the rotational motion at high excitation to be intuitively understood: Each quantized energy level corresponds to a trajectory on the RES which is directly related to the motion in space.

We have already pointed out that at present, our energy results are not accurate enough to be of use to high-resolution molecular spectroscopists, but we hope that future, more detailed investigations of the vibrationally averaged, TROVE-generated RES might lead to new insights into the accuracy achievable by the semi-classical approach. The present work has shown that at the moment, the semi-classical approach with a RES generated from a Watson-type effective rotational Hamiltonian produces energies deviating from their fully quantum-mechanical counterparts by about 2 cm^–1^ on the average. At high *J* values, where the correspondence principle is valid, a similar level of agreement is obtained between the TROVE-RES semi-classical energies and the quantum-mechanical results (Fig. 4 and 5). This is one of the chief results of the present work: Using the variational TROVE approach to generate a RES for further use in a semi-classical analysis of the rotational energy levels for various vibrational states is possible and produces energies with a relative deviation from exact quantum calculations of a few percent. This is – to our knowledge – the first time such a combined approach of variational principle and semi-classical analysis has been employed for molecular dynamics.

From a physical point of view, we think that a more thorough investigation of the interplay between vibrational states and their respective RESs is the key to describing the molecular motion at high energy. In the highly excited states located there, rotation-vibration coupling is very important since the interaction of a given state with increasingly higher excited vibrational states has to be taken into account. Since we have reformulated the theory in terms of Gutzwillers trace formula,^42^ another interesting issue would be a semi-classical treatment of rotational and vibrational degrees of freedom simultaneously, because Gutzwiller's formula can be generalized to higher dimensions. Hence, we think, a comparison to the methods used, *e.g.*, by Frederick *et al.*,^47^ where one bending vibration has been incorporated with the semi-classical rotation, would be fruitful. We expect a scheme, analogous to that of decoupled rotations described in the present work, to be appropriate also in this case.
